# The role of inflammation, the autonomic nervous system and classical cardiovascular disease risk factors on subendocardial viability ratio in patients with RA: a cross-sectional and longitudinal study

**DOI:** 10.1186/ar4103

**Published:** 2012-11-28

**Authors:** Aamer Sandoo, Athanassios D Protogerou, James Hodson, Jacqueline P Smith, Evi Zampeli, Petros P Sfikakis, George D Kitas

**Affiliations:** 1 Department of Rheumatology, Dudley Group of Hospitals NHS Trust, Russells Hall Hospital, Dudley DY1 2HQ, UK; 2 School of Sport and Exercise Sciences, University of Birmingham, Edgbaston, Birmingham B15 2TT, UK; 3 First Department of Propaedeutic and Internal Medicine, University Medical School, Athens, Greece; 4 Wolfson Computer Laboratory, University Hospital Birmingham NHS Foundation Trust, Queen Elizabeth Hospital Birmingham, Mindelsohn Way, Birmingham B15 2WB, UK; 5 Arthritis Research UK Epidemiology Unit, University of Manchester, Oxford Road, Manchester M13 9PT, UK

## Abstract

**Introduction:**

Evidence indicates that rheumatoid arthritis (RA) patients have increased susceptibility to myocardial ischaemia that contributes to myocardial infarction. The subendocardial viability ratio (SEVR) can be measured using pulse wave analysis and reflects myocardial oxygen supply and demand. The objective of the present study was to examine specific predictors of SEVR in RA patients, with a specific focus on inflammation and classical cardiovascular disease (CVD) risk factors.

**Methods:**

Two patient cohorts were included in the study; a primary cohort consisting of 220 RA patients and a validation cohort of 127 RA patients. All patients underwent assessment of SEVR using pulse wave analysis. Thirty-one patients from the primary cohort who were about to start anti-inflammatory treatment were prospectively examined for SEVR at pretreatment baseline and 2 weeks, 3 months and 1 year following treatment. Systemic markers of disease activity and classical CVD risk factors were assessed in all patients.

**Results:**

The SEVR (mean ± standard deviation) for RA in the primary cohort was 148 ± 27 and in the validation cohort was 142 ± 25. Regression analyses revealed that all parameters of RA disease activity were associated with SEVR, along with gender, blood pressure and heart rate. These findings were the same in the validation cohort. Analysis of longitudinal data showed that C-reactive protein (*P *< 0.001), erythrocyte sedimentation rate (*P *< 0.005), Disease Activity Score in 28 joints (*P *< 0.001), mean blood pressure (*P *< 0.005) and augmentation index (*P *< 0.001) were significantly reduced after commencing anti-TNFα treatment. Increasing C-reactive protein was found to be associated with a reduction in SEVR (*P *= 0.02) and an increase in augmentation index (*P *= 0.001).

**Conclusion:**

The present findings reveal that the SEVR is associated with markers of disease activity as well as highly prevalent classical CVD risk factors in RA, such as high blood pressure and diabetes. Further prospective studies are required to determine whether the SEVR predicts future cardiac events in RA.

## Introduction

Rheumatoid arthritis (RA) is a chronic inflammatory musculoskeletal disease that affects ~0.8% of the adult population. RA also associates with an increased risk for cardiovascular disease (CVD) [1], which is only partially explained by traditional CVD risk factors [2]. The inflammatory processes of RA and CVD are remarkably similar, suggesting that RA itself may be an important risk factor for CVD [3].

The leading cause of cardiac death in RA is myocardial infarction [4]. A study in RA patients without overt coronary artery disease reported abnormalities in myocardial perfusion using contrast-enhanced magnetic resonance imaging (cMRI), and the degree of abnormality was related to the extent of RA disease-related inflammation [5]. In another study, RA patients undergoing coronary angiography had greater myocardial ischaemia than healthy controls who were matched for classical CVD risk factors, but had similar levels of ischaemia to age-matched and sex-matched diabetic controls [6]. Furthermore, myocardial ischaemia was evident despite the absence of obstructive coronary artery disease, suggesting a microvascular cause for the ischaemia [6].

The current gold standard measure of microvascular coronary perfusion is coronary angiography; however, this procedure is invasive, carries considerable risk to the patient and cannot be repeated at regular intervals. Arterial tonometry using pulse wave analysis has recently gained interest in clinical research as it can non-invasively measure the central aortic pressure waveform and yield information on the augmentation index (AIx) (arterial stiffness), ejection duration (duration of ventricular contraction), pulse pressure (PP; pressure generated by the left ventricle during systole), mean blood pressure (MBP; product of cardiac output and systemic vascular resistance), tension time index (TTI; pressure load during systole), diastolic time index (DTI; pressure load during diastole) and subendocardial viability ratio (SEVR; a measure of coronary microvascular perfusion) [7].

In particular, the SEVR reflects myocardial oxygen supply and demand, with low values representing a lesser degree of myocardial perfusion [8]. The ratio of DTI versus TTI (that is, the integral of pressure and time during diastole and systole, respectively) has been shown to correlate well with the ratio of subepicardial to subendocardial blood flow, and therefore represents an index of subendocardial viability, defined as the SEVR (Buckberg index) [9,10]. The SEVR is an independent predictor of coronary flow reserve in patients with essential hypertension [11], and is related to coronary artery calcification in patients with diabetes [12]. In addition, low SEVR associates with severity of diabetes [13], poor renal function [14], low physical fitness [15], and cigarette smoking [16]. As such, the SEVR can be utilised as a surrogate measure of coronary microvascular perfusion in a number of different clinical populations.

To our knowledge only two studies have examined the SEVR in patients with RA [17,18]; one small pilot study revealed that the SEVR improved following 12 months of lipid-lowering treatment when compared with a group receiving only a placebo drug [17], while in another study the SEVR was worse following 7 weeks of anti-inflammatory treatment in RA and ankylosing spondylitis patients [18]. However, the small sample sizes of both studies, as well as the inclusion of different patient groups in the latter study, make these findings difficult to interpret.

The aims of the present study were: to examine associations between disease-related inflammation and classical CVD risk factors with SEVR in two separate cohorts of RA patients; and to prospectively assess the long-term effects of anti-inflammatory treatment on SEVR.

## Materials and methods

### Patients

Two hundred and twenty consecutive RA patients were recruited from the rheumatology outpatient clinics of the Dudley Group of Hospitals NHS Trust, UK and formed the primary cohort. A separate validation cohort consisting of 127 consecutive RA patients were recruited from the rheumatology outpatient clinics of the First Department of Propaedeutic and Internal Medicine, Laikon Hospital, Greece. All patients met the retrospective application of the 1987 revised RA criteria of the American College of Rheumatism [19]. Patients were excluded if they had previously confirmed acute coronary syndrome, established CVD or serious psychiatric disorder as indicated in their medical notes and/or on questioning during the initial consultation. The study received ethics approval from Birmingham, East, North and Solihull Research Ethics Committee and all participants gave their written informed consent according to the Declaration of Helsinki.

### Protocol

Patients reported to a temperature-controlled vascular laboratory (22 to 24°C) after a 12-hour overnight fast. Patients from both cohorts underwent a detailed clinical examination, which included evaluation of their medical history and hospital records, and assessment of their height and weight, as well as calculation of their body mass index. In addition, demographic information was collected from all patients by questionnaire. The Disease Activity Score in 28 joints (DAS28) [20] and the Anglicised version of the Stanford Health Assessment Questionnaire [21] were completed in the primary cohort only. Blood pressure measurements were taken using an automated blood pressure monitor - Datascope Accutor (Montvale, New Jersey, USA) and Microlife WatchBP Office (Widnau, Switzerland) for the primary and validation cohorts, respectively - after the patients had rested for 20 minutes. A blood sample was also obtained. Following this, the participants underwent assessment of the SEVR using pulse wave analysis.

### Blood sampling

Serum from patients in the primary cohort was analysed for total cholesterol, high-density lipoprotein cholesterol, triglycerides, C-reactive protein (CRP) and glucose using a Vitros^® ^5.1 FS Chemistry system (Ortho Clinical, High Wycombe, Bucks, UK). The Westergren erythrocyte sedimentation rate (ESR) was measured using the Starrsed Compact (Mechatronics BV, Hoorn, the Netherlands). All tests were carried out in the routine and research laboratories of Russells Hall Hospital, Dudley Group NHS Foundation Trust, UK. In the validation cohort, only CRP was measured using a Vitros^® ^5.1 FS Chemistry system in the routine laboratory of Laikon Hospital, Greece.

### Pulse wave analysis

Pulse wave analysis (SphygmoCor Px Pulse Wave Analysis; ScanMed Medical Instruments, Moreton in Marsh, UK) was used to determine the SEVR, AIx, PP, MBP, TTI, and DTI. After recording the brachial blood pressure, the left radial artery was palpated to identify a suitable pulse. The applanation tonometer was positioned over the artery with enough pressure to flatten (but not occlude) the patient's radial artery. The applanation tonometer detects the PP wave, which is calibrated against the standard brachial blood pressure and gives the maximum (systolic) and minimum (diastolic) points of the pressure wave. The pressure waveform is then mathematically transformed into a central aortic waveform that contains the first (peak flow) and second (peak pressure) systolic peaks and displays information on ejection duration.

The SEVR is calculated by utilising the ejection duration that is automatically measured by the Sphygmocor system and dividing the area under the diastolic curve (coronary perfusion) by the area under the systolic curve (cardiac workload). In normal conditions, the SEVR can be anywhere between 130 and 200%. Values that are below unity (100%) reflect poor perfusion of the subendocardium [8]. The AIx is calculated as the difference between the second and first systolic peaks and is expressed as a percentage of the PP, with a high value indicating greater arterial stiffness. The pressure waveforms in the radial artery were recorded for an 11-second period. The software integrated in the analyser displayed an operator index that reflects the quality of the recorded waveform. If the operator index was low (< 75), another reading was taken. Three readings with an operator index > 75 were used for analysis. For each parameter, the average of three readings was calculated. Owing to some loss of data, PP was only available for 147 patients from the primary cohort.

### Longitudinal study cohort

Thirty-one patients from the primary cohort who were about to start anti-inflammatory treatment (anti-TNFα, *n *= 21), intravenous glucocorticoids (*n *= 6), and rituximab (*n *= 4) on clinical indication (UK guidelines) were recruited into a 1-year prospective study. All of the assessments described above were conducted before patients started treatment (pretreatment baseline), and repeated at 2 weeks, 3 months and 1 year after initiation of treatment. For patients starting on anti-TNFα, assessments at 2 weeks and 3 months were performed before receiving the next dose of their anti-TNFα drug.

### Statistical analysis

#### Cross-sectional study

Statistical analysis was performed using SPSS18 (SPSS Inc., Chicago, IL, USA). Variables were tested for normality by the Kolmogorov-Smirnov test. Log transformation was performed for positively skewed variables as appropriate. Values are expressed as the mean (standard error), geometric mean (95% confidence interval), median (25th to 75th percentile values) or percentages, as appropriate. Univariate analysis of variance was used to assess differences in the SEVR (dependent variable) according to gender, presence of CVD risk factors, medication use and rheumatoid factor positivity (fixed factors). To identify specific determinants of the SEVR in RA, linear regression (continuous variables) and logistic regression (discontinuous variables) were used. The SEVR was entered as the dependent variable, and demographic, CVD risk factors and inflammatory variables were entered separately as independent variables.

#### Longitudinal study

The longitudinal data were analysed using generalised estimating equations. This allowed for the potential correlations between repeated measurements made on the same patients to be adjusted for. To consider the effect of the anti-inflammatory treatment, separate models were produced for each of the outcomes considered, which were log_10_-transformed prior to the analysis, where applicable. The measurement time was entered as a factor in each model, with the baseline value set as the reference category. An unstructured correlation structure was used where possible, since this is the most general option, with exchangeable structures used where convergence was not achieved.

CRP and the ESR were then included separately as factors in these models. This separate inclusion allowed for the effect each of these factors on the other outcomes to be considered, whilst adjusting for the changes over time.

## Results

### General characteristics

The patient characteristics for both cohorts are presented in Table 1. The majority of patients were female and had low to moderate disease activity levels.

**Table 1:** Characteristics and pulse wave analysis for rheumatoid arthritis patients in both cross-sectional and longitudinal cohorts

	Rheumatoid arthritis patients		
	
	Primary cohort (*n *= 220)	Validation cohort (*n *= 127)	Longitudinal cohort (*n *= 31)
Age (years)	61 ± 12	59 ± 13	53 ± 13
Sex female	176 (80)	103 (81)	21 (68)
Disease-related characteristics			
Disease duration (years)	17 ± 12	10 ± 10	13 ± 13
Rheumatoid factor-positive (%)	126 (79)	n/a	23 (74)
Erythrocyte sedimentation rate (mm/hour)	12 (7 to 26)	22 (12 to 38) (*n *= 112)	10 (4 to 16)
C-reactive protein (mg/l)	3 (3 to 12)	5 (3 to12)	18 (9 to 33)
DAS28	3.29 ± 1.26	n/a	4.32 ± 0.87
HAQ	1.6 ± 0.9	n/a	2.19 ± 0.42
Classical CVD risk factors			
Body mass index	29 ± 6	33 ± 12	29 ± 6
Current smokers	33 (15)	40 (31)	7 (23)
Diabetes mellitus	17 (8)	10 (8)	0 (0)
Dyslipidaemia	60 (27)	30 (24)	7 (23)
SBP (mmHg)	134 ± 18	134 ± 18	130 ± 14
DBP (mmHg)	79 ± 11	79 ± 10	81 ± 11
Heart rate (beats/minute)	74 ± 13	68 ± 11	73 ± 12
Total cholesterol (mmol/l)	5.0 ± 1.0	5.2 ± 0.9 (*n *= 114)	5.0 ± 0.9
HDL (mmol/l)	1.5 ± 0.4	1.6 ± 0.7 (*n *= 102)	1.5 ± 0.3
Triglycerides (mmol/l)	1.3 ± 0.6	1.2 ± 0.4 (*n *= 95)	1.5 ± 0.6
TC:HDL ratio	3.5 ± 1.1	3.5 ± 0.8 (*n *= 102)	3.5 ± 0.8
RA disease-specific medications			
Methotrexate	124 (65)	76 (60)	19 (61)
Sulfasalazine	44 (24)	0	5 (16)
Hydroxychloroquine	50 (28)	0	4 (13)
Leflunomide	15 (9)	16 (13)	0 (0)
Prednisolone	48 (26)	85 (67)	6 (19)
NSAID	34 (20)	3 (2)	6 (19)
COX II inhibitors	12 (7)	0 (0)	2 (7)
Analgesic	74 (40)	n/a	8 (26)
Folic acid	118 (63)	63 (50)	16 (52)
Anti-TNFα	44 (20)	51 (40)	--
CVD medications			
Anti-hypertensive	67 (36)	68 (66)	6 (19)
Anti-hypercholesterolemics	57 (31)	23 (18)	4 (13)
Beta-blocker	20 (11)	28 (22)	2 (7)
Calcium channel-blocker	24 (14)	19 (15)	0 (0)
Pulse wave analysis			
Ejection duration (m/second)	312 ± 27	331 ± 26	320 ± 25
Pulse pressure (mmHg)^b^	46 ± 13^a^	54 ± 14	-
Mean pressure (mmHg)	99 ± 12	97 ± 11	99 ± 10
Tension time index	2,424 ± 402	2,485 ± 441	2,359 ± 368
Diastolic time index	3,493 ± 462	3,443 ± 472	3,523 ± 651
SEVR (%)	148 ± 27	142 ± 25	157 ± 33
Augmentation index (%)	33 ± 9	34 ± 10	32 ± 9

Results expressed as number (percentage), median (25th to 75th percentile values) or mean ± standard deviation. COX, cyclooxygenase; CVD, cardiovascular disease; DAS28, Disease Activity Score in 28 joints; DBP, diastolic blood pressure; HAQ, Health Assessment Questionnaire; HDL, high-density lipoprotein; n/a, not available; NSAID, nonsteroidal anti-inflammatory drug; SBP, systolic blood pressure; SEVR, subendocardial viability ratio; TC, total cholesterol. ^a^Data only available for 147 patients. ^b^Data unavailable for longitudinal cohort.

### Effects of medications on the subendocardial viability ratio

Univariate analysis of variance showed that the SEVR did not differ between patients receiving or not receiving steroid treatment in both the primary and validation cohorts. This was a similar finding for disease-modifying anti-rheumatic drug use, biologic agents, antihypertensives, anti-hypercholesterolemics, nonsteroidal anti-inflammatory drugs, and cyclooxygenase II inhibitors. However, the SEVR was higher in patients receiving β-blockers in the primary cohort only (167 ± 24 vs. 146 ± 27, *P *< 0.01).

### Effects of CVD risk factors on the subendocardial viability ratio

Univariate analysis of variance was used to compare SEVR levels in patients with and without classical CVD risk factors. This analysis revealed that patients with diabetes had significantly lower SEVR than nondiabetic patients (127 ± 19 and 149 ± 26 respectively, *P *< 0.001). There was no difference in SEVR for any of the other CVD risk factors. Similar findings were also apparent in the validation cohort, except that the SEVR between diabetic and nondiabetic patients did not reach statistical significance (133 ± 31 and 142 ± 24 respectively, *P *= 0.28).

### Univariate predictors of the subendocardial viability ratio

Linear and binary regression analysis was performed to determine univariate predictors (independent variable) of the SEVR (dependent variable) in patients with RA (see Table 2). This analysis revealed that gender was associated with the SEVR in the primary cohort, with females having lower SEVR than males (144 ± 25 and 162 ± 28 respectively, *P *< 0.001). Similarly, gender was also associated with the SEVR in the validation cohort; with females having a lower SEVR than males (139 ± 25 and 151 ± 24 respectively, *P *= 0.04). From the disease-related variables, log ESR and log CRP were inversely associated with the SEVR, and a positive association was found with log disease duration in patients in the primary cohort (Table 2). Only PP and heart rate were inversely associated with the SEVR from the classical CVD risk factors. However, there were trends for an inverse association between body mass index, total cholesterol and SEVR. In the validation cohort, log CRP (but not disease duration and ESR) was inversely associated with the SEVR (Table 2). As with the primary cohort, PP (marginally MBP) and heart rate were the CVD risk factors associated with the SEVR.

**Table 2:** Regression analysis between general characteristics, disease-related characteristics, classical cardiovascular disease risk factors and the SEVR

	Primary cohort	Validation cohort
General characteristics		
Age	*β = 0*.02, *P *= 0.79	*β = *0.03, *P *= 0.73
Gender	0.97 (0.96 to 0.99)*	0.98 (0.96 to 0.99)*
Disease-related characteristics		
Log disease duration	***β = *0.21, *p *= 0.01**	*β = *0.12, *P *= 0.21
RF positivity	0.90 (0.99 to 1.02)	n/a
Log ESR	***β = -*0.20, *P *= 0.01**	*β*= -0.144, *P *= 0.131
Log CRP	***β = -*0.23, *P *= 0.003**	***β = -*0.28, *P *= 0.002**
DAS28	*β = -*0.12, *P *= 0.09	n/a
HAQ	*β = -*0.11, *P *= 0.16	n/a
Classical CVD risk factors		
Body mass index	*β = - *0.13, *P *= 0.05	*β = - *0.19, *P *= 0.18
Pulse pressure	***β = -*0.23, *P *= 0.004**	***β = - *0.21, *P *= 0.017**
Mean blood pressure	*β = -*0.10, *P *= 0.15	*β = -*0.17, *P *= 0.05
Heart rate	***β = *-0.64, *P *< 0.001**	***β = -*0.76 *P *< 0.001**
Total cholesterol	*β = -*0.12, *P *= 0.09	*β = -*0.14, *P *= 0.14
TC:HDL ratio	*β = -*0.02, *P *= 0.76	*β = -*0.09, *P *= 0.35
Triglycerides	*β = -*0.01, *P *= 0.90	*β = *0.01, *P *= 0.94

Data presented as *β *and *P *values or odds ratio (95% confidence interval). CRP, C reactive protein; CVD, cardiovascular disease; DAS28, Disease Activity Score in 28 joints; ESR, erythrocyte sedimentation rate; HAQ, Health Assessment Questionnaire; HDL, high density lipoprotein; n/a, not available; RF, rheumatoid factor; SEVR, subendocardial viability ratio; TC, total cholesterol. *P < 0.001. Bold data represent statistically significant results.

### Multivariate predictors of the subendocardial viability ratio in the primary cohort

Stepwise multivariate regression was performed to examine the relationship between significant univariate predictors presented in the analysis above (independent variables) and the SEVR (dependent variables). The individual components that were entered into the model were gender, log disease duration, log ESR, log CRP, PP and heart rate. The entry probability was 0.05 and none of the variables were forced back into the model. From this analysis a significant model emerged (adjusted *R*^2 ^= 0.631, *F*_4, 76 _= 35.3, *P *< 0.001). Significant variables within the model included heart rate (*β = *-0.75, *P <*0.001), PP (*β *= -0.39, *P <*0.001), log ESR (*β *= -0.29, *p *= 0.001) and log CRP (*β *= 0.18, *P *= 0.03).

### Multivariate predictors of the subendocardial viability ratio in the validation cohort

Similar multivariate regression as above was performed in the validation cohort. The individual components that were entered into the model as independent variables were gender, log CRP, PP and heart rate, with the SEVR entered as the dependent variable. From this analysis a significant model was apparent (adjusted *R*^2 ^= 0.69, *F *= 72.1, *P *< 0.001). Significant variables within the model included heart rate (*β = *-0.783, *P <*0.001), PP (*β = *-0.258, *P <*0.001), and gender (*β = *-0.249, *P *< 0.01). In an alternative model without heart rate, log CRP was, independently of gender and PP, associated with the SEVR (*β = *-0.238, *P <*0.05).

### Inflammation and components of the subendocardial viability ratio

To examine the effects of inflammation on the components used to calculate the SEVR, further linear regression analysis was performed with DTI, TTI and heart rate entered separately as dependent variables, and log ESR, log CRP as well as DAS28 entered separately as independent variables. This analysis showed that DTI was inversely associated with all parameters of disease activity, but no such associations were apparent for TTI (see Table 3). Heart rate was inversely associated with log ESR and log CRP only. In the validation cohort, TTI was positively associated with log CRP (*β *= 0.28, *P *= 0.002) while heart rate was inversely associated with log CRP (*β *= -0.27, *P *= 0.01) (Table 3).

**Table 3:** Regression analysis between individual components of the SEVR and inflammation in the primary cohort

	Log ESR	Log CRP	DAS28
Primary cohort			
DTI	***β = -*0.26, *P *= 0.001**	***β = -*0.17, *P *= 0.03**	***β = -*0.20, *P *= 0.004**
TTI	*β = *0.02, *P *= 0.85	*β* = 0.13, *P *= 0.08	*β = -*0.006, *P *= 0.38
HR	***β = *0.18, *P *= 0.02**	***β = *0.32, *P *< 0.001**	*β = *0.13, *P *= 0.006
Validation cohort			
DTI	*β = -*0.02, *P *= 0.81	*β = *-0.053, *P *= 0.56	n/a
TTI	*β = *0.16, *P *= 0.10	***β = *0.28, *P *= 0.002**	n/a
HR	*β = *0.85, *P *= 0.37	***β = -*0.27, *P *= 0.01**	n/a

CRP, C-reactive protein; DAS28, Disease Activity Score in 28 joints; DTI, diastolic time index; ESR, erythrocyte sedimentation rate; HR, heart rate; n/a, not available; SEVR, subendocardial viability ratio; TTI, tension time index. Bold data represent statistically significant results.

### Longitudinal study

The effect of anti-inflammatory treatment on a range of outcomes was considered using generalised estimating equations (Table 4). Each outcome was considered separately, with the time that measurements were taken included as a categorical factor. This analysis gave an overall significance for the change over time, which tested the null hypothesis that the outcome considered remained constant for the year after the commencement of treatment. There was significant evidence against this hypothesis for CRP (*P *< 0.001), ESR (*P *= 0.004), DAS28 (*P *< 0.001), MBP (*P *= 0.002) and AIx (*P *< 0.001), with the only nonsignificant outcome being the SEVR (*P *= 0.30). Hence, for those outcomes that were significant, it can be concluded that the average measurements changed over time.

**Table 4:** Generalised estimating equations testing the effect of time on various measurements.

	CRP^a^	ESR^a^	DAS28	SEVR^a^	MBP^a^	AIx
Significance of change over time						
*P *value	**< 0.001**	**0.004**	**< 0.001**	0.30	**0.002**	**< 0.001**
Baseline						
Mean^b^	8.7 (5.7 to 13.2)	16.8 (11.3 to 25.0)	4.3 (0.17)	153.3 (142.1 to 165.5)	98.9 (95.2 to 102.8)	31.9 (1.56)
2 weeks						
Mean^b^	4.2 (2.8 to 6.4)	10.8 (7.4 to 15.9)	2.8 (0.26)	154.8 (144.9 to 165.3)	94.4 (90.2 to 98.8)	30.2 (1.74)
*P *value^c^	**< 0.001**	**< 0.001**	**< 0.001**	0.69	**0.010**	0.11
3 months						
Mean^b^	5.5 (3.7 to 8.3)	11.4 (6.9 to 18.9)	2.8 (0.27)	160.4 (149.6 to 172.0)	92.9 (88.0 to 98.1)	32.3 (1.98)
*P *value^c^	**< 0.001**	0.05	**< 0.001**	0.14	**0.003**	0.75
1 year						
Mean^b^	2.8 (1.6 to 5.1)	11.9 (7.9 to 17.7)	2.6 (0.29)	154.1 (140.5 to 169.1)	92.7 (87.3 to 98.5)	29.6 (2.15)
*P *value^c^	**< 0.001**	0.33	**< 0.001**	0.85	**0.002**	0.13

AIx, augmentation index; CRP, C-reactive protein; ESR, erythrocyte sedimentation rate; DAS28, Disease Activity Score in 28 joints; MBP, mean blood pressure; SEVR, subendocardial viability ratio. From a generalised estimating equation, with unstructured correlation structure. ^a^Log_10_-transformed prior to analysis. ^b^Data displayed as geometric mean (95% confidence interval) or mean (standard error) as applicable. ^c^Versus baseline. Bold data represent statistically significant results.

To detect where these changes occurred, pairwise comparisons were made between the baseline and all of the subsequent measurement times. Where this was significant, it implied that the outcome considered changed significantly after the treatment began. As can be seen in Table 4, in each case that a pairwise comparison was significant, the observed mean was smaller than that at baseline; hence, the treatment brought about a significant reduction in these outcomes.

A secondary analysis considered the effect of CRP and ESR on the other factors considered above (Table 5). Generalised estimating equations were again used to account for the potential correlation between repeated measurements made on the same patient. In each model, either the CRP or ESR was entered as a continuous predictor, with the SEVR, MBP or AIx as the dependent variable. The resulting *P *value then represented the strength of the relationship between the two variables in the model. The coefficient demonstrated the increase in the dependent variable that would be expected for each increase in the predictor variable. Owing to the necessity of log transformations, this relationship was not necessarily linear.

**Table 5:** Generalised estimating equations testing effect of CRP and ESR on SEVR, MBP and augmentation index

Outcome	Effect of CRP^a^		Effect of ESR^a^	
	Coefficient (95% CI)	*P *value	Coefficient (95% CI)	*P *value
SEVR^a^	-0.031 (-0.059 to -0.004)	**0.02**	-0.028 (-0.079 to 0.023)	0.28
MBP^a^	0.009 (-0.009 to 0.026)^b^	0.34^b^	-0.009 (-0.033 to 0.015)^b^	0.48^b^
AIx	5.434 (1.769 to 7.299)	**0.001**	-1.887 (-4.877 to 1.104)	0.22

AIx, augmentation index; CI, confidence interval; CRP, C-reactive protein; ESR, erythrocyte sedimentation rate; MBP, mean blood pressure; SEVR, subendocardial viability ratio. From a generalised estimating equation, with unstructured correlation structure, unless stated otherwise. ^a^Log_10_-transformed prior to analysis. ^b^Exchangeable correlation structure used.

The ESR was not found to be a significant predictor of any of these outcomes, whilst CRP was found to have significant effects on both the AIx (*P *< 0.001) and the SEVR (*P *= 0.02). For the former, the resulting coefficient implied that a 10-fold increase in CRP increases the AIx by 5.4 (95% confidence interval = 1.8 to 7.3). Because both of these variables are log_10 _transformed, the relationship between CRP and the SEVR is more complex. The model showed that a 10-fold increase in CRP reduces the log_10 _of the SEVR by 0.03 (95% confidence interval = 0.00 to 0.06). Applying this to a real-world example, an increase of CRP from 1 to 10 would be expected to reduce the SEVR from 174 to 162. A 10-fold increase in CRP hence reduces the SEVR by approximately 12.

## Discussion

The present findings from two separate cohorts of RA patients revealed that SEVR, a non-invasive measure of subendocardial perfusion, is associated with disease-related inflammation along with classical CVD risk factors such as the heart rate, PP and diabetes. In particular, these data generate the hypothesis that inflammation impairs myocardial perfusion, as assessed by the SEVR, via the stimulation of the autonomic nervous system; that is, increased heart rate and especially a decrease in DTI. Alternatively, as suggested by the validation cohort data, the SEVR may also be reduced due to increased TTI in the presence of higher inflammation.

Coronary artery disease is typically silent in RA, with a greater frequency of unrecognised myocardial infarction and sudden cardiac death [22]. This highlights the importance of identifying myocardial perfusion defects as early as possible. Assessment of the SEVR is non-invasive, simple to perform, carries no risk to the participant and can be repeated at regular intervals. The SEVR primarily reflects diastolic dysfunction and plays an important role in the development of CVD [23], and associates with invasive measures of coronary microvascular function such as coronary flow reserve [11]. In addition, the SEVR is related to a number of clinical conditions including diabetes [13], impaired renal function [14], low physical fitness [15], and cigarette smoking [16]. Assessment of the SEVR thus appears to be a valid surrogate measure of coronary microvascular perfusion, particularly in a research setting with healthy volunteers and in patients without overt CVD. In a 2-year prospective study conducted by Protogerou and colleagues in frail older participants exhibiting the blood pressure J-curve phenomenon (that is, presenting higher mortality in the presence of low diastolic blood pressure), the SEVR was significantly impaired in the higher mortality group - providing further evidence that an impaired SEVR could have an impact on CVD, independently from other classical markers of cardiovascular health (such as intima-media thickness, pulse wave velocity, left ventricular mass - all of which were similar between the groups) [24].

The inflammatory nature of RA and CVD is strikingly similar [3], and on this basis it has been hypothesised that RA disease-related inflammation contributes to the excess CVD risk present in RA [25]. The present study examined associations between disease-related inflammation and the SEVR in a large cohort of patients and demonstrated that the SEVR was associated with all markers of disease activity and inflammation (DAS28, ESR and CRP). Furthermore, although there was no evidence that SEVR changed significantly in response to anti-inflammatory treatment in the longitudinal study, the latter analysis found a significant negative relationship between CRP and SEVR (*P *= 0.02). The results of the model showed that a 10-fold increase in CRP is associated in a reduction of 0.031 in the log_10 _SEVR. To our knowledge only one other study, with a small sample size of RA patients (*n *= 17), has assessed the effects of inflammation on the SEVR in RA, and revealed that the SEVR was worse following 7 weeks of treatment with anti-TNFα despite a reduction in disease activity and inflammation [18]. However, the findings of that study need to be interpreted with caution as the treatment group also consisted of 13 ankylosing spondylitis patients, and analysis was performed by categorising the RA patients and ankylosing spondylitis patients into one group. In addition, the reduction in SEVR following treatment (ΔSEVR 7.4%) was smaller than the nonsignificant difference in SEVR between patients and healthy controls at baseline (ΔSEVR 10.1%), thus limiting the clinical relevance of this finding [18].

Studies assessing coronary microvascular function consistently demonstrate inflammation to be an important contributor to myocardial ischaemia. For example, Kobayashi and colleagues demonstrated myocardial perfusion defects in RA patients using cMRI [5]. Furthermore, they reported that abnormal cMRI was associated with higher DAS28, and patients with impaired cMRI had significantly higher levels of ESR and CRP than patients with normal cMRI [5]. Another study in RA revealed that myocardial perfusion defects were as a result of abnormalities in the coronary microvasculature and that this was associated with high disease activity [6]. These cross-sectional findings suggest that inflammation might more readily affect microvascular function, rather than macrovascular function, but further prospective studies specifically examining this hypothesis are warranted.

In the present work, inflammation was inversely associated with DTI and positively associated with heart rate. These findings suggest that inflammation may lower the time for diastolic filling of the heart while simultaneously increasing cardiac workload due to an elevated heart rate. Both of these actions would have a negative impact on cardiac perfusion and contribute to myocardial ischaemia, especially as the heart rate was inversely associated with the SEVR in multivariate analysis. Inflammation therefore appears to have a detrimental association with myocardial ischaemia, possibly due to negative actions on cardiac perfusion and workload. Alternatively, as shown in the validation cohort, higher CRP levels may be associated with lower SEVR due to increased TTI (that is, afterload (PP)), presumably induced by the described effect of inflammation and heart rate acceleration on aortic stiffness.

Another important finding in the current study was that the SEVR was positively associated with disease duration. Patients with greater disease duration therefore had better myocardial perfusion. This finding appears to contradict the general hypothesis that longer disease duration is associated with greater CVD risk [26]. However, a number of studies have revealed an increased risk for myocardial infarction within 1 year of RA diagnosis [22,27], and increased risk of hospitalisation from CVD occurs within 7 years of RA diagnosis [28]. Furthermore, studies utilising other surrogate measures of coronary endothelial function have revealed that changes in flow-mediated dilatation appear to be already evident within 1 year of RA diagnosis [29], but do not appear to be further influenced by disease duration [30,31]. Similarly, carotid artery atherosclerosis is apparent even in patients with a recent diagnosis of RA [32,33] - and while some studies have found an association between carotid artery atherosclerosis and longer disease duration [34,35], other studies have not [36,37]. Collectively, these studies suggest that increased CVD risk is evident early in the disease course, with reduced risk with longer disease duration. One possible explanation for these findings could be that optimal treatment strategies that effectively control RA symptoms as well as CVD risk are more likely to be achieved with longer disease duration, and as a result the patient will have more stable disease, better myocardial perfusion and lower CVD risk. Further prospective studies that assess the SEVR in patients newly diagnosed with RA and who are followed over the course of their disease are warranted.

The SEVR was found to inversely associate with PP in the present cohort, but not with MBP. This finding supports the results of a meta-analysis which revealed that PP but not MBP was an independent predictor of cardiovascular complications and all-cause mortality in older hypertensive patients [38]. PP is generated by the left ventricle during systole and is dampened by the compliance of the aorta. Loss of aortic compliance with age or CVD will lead to greater PPs, and there is evidence that arterial stiffness is increased in RA [39]. In addition, conditions such as hypertension can increase afterload (the pressure the left ventricle has to overcome to eject blood into the systemic circulation), which results in increased left ventricular workload and consequently greater myocardial oxygen demand, increasing the susceptibility for myocardial ischaemia [40]. The prevalence of hypertension in RA is high and the condition is often suboptimally treated [41]. In the present cohort, 44% of patients were hypertensive but the SEVR did not differ between hypertensive and nonhypertensive patients. This lack of difference is possibly because the majority of hypertensive patients were on angiotensin-converting enzyme or angiotensin II subtype-1 receptor inhibitors as well as β-blockers. Such treatment increases myocardial perfusion and decreases myocardial workload [42], hence making a difference in the SEVR between hypertensive and nonhypertensive patients unlikely. This is supported by our finding that the SEVR was better in patients receiving β-blockers (compared with those who were not).

RA patients with diabetes had lower SEVR than nondiabetic patients, in line with findings from another study in type 1 diabetics [13]. A number of factors, including increased glucose levels, can perpetuate damage to the vasculature in diabetic patients [43], but there is growing evidence that autonomic dysfunction can adversely alter heart rate and vascular tone, thereby contributing to myocardial ischaemia in diabetes [16,44,45]. Importantly, there is also evidence for autonomic dysfunction in RA [46], with inflammation being a potential mediating factor [47]. Given that RA and diabetes have a similar CVD risk burden [48] and vascular profile [49], further research examining the role of cardiac autonomic neuropathy on multiple cardiovascular parameters (including the SEVR) is required.

The strength of the present study is the inclusion of two separate cohorts of well-characterised RA patients along with the assessment of a novel and non-invasive measure of myocardial ischaemia. In addition, the 1-year prospective study allowed observation of the effect of anti-inflammatory treatment on the SEVR. Importantly, the findings were replicated in both cohorts, demonstrating the strength of the findings. The limitations of the study include the prospective assessment of the SEVR following administration of three different types of anti-inflammatory treatment (anti-TNFα, intravenous corticosteroids and rituximab) whose mode of action could differentially affect the vasculature. However, there was no difference in parameters of pulse wave analysis between treatment types at any time point (data not shown). Another limitation in the prospective arm of the study was that it was not possible to determine whether parameters of pulse wave analysis were already impaired at baseline due to an absence of a healthy control group. Nevertheless, the average AIx relative to the age of the prospective cohort would be considered normal, and the AIx did improve with treatment at 1 year - which tends to suggest that irreversible vascular alterations were not evident in this cohort. Finally, the prospective arm of the study was possibly underpowered to detect a change in SEVR following treatment; further prospective studies with a large sample size as well as including a more thorough examination of the factors that affect the ventricular-vascular coupling are therefore warranted.

## Conclusion

In summary, the present study reveals that systemic markers of inflammation and some classical CVD risk factors associate with myocardial ischaemia, but further prospective studies that assess whether the SEVR predicts future cardiac events in RA and other populations are warranted.

## Abbreviations

AIx: augmentation index; CRP: C-reactive protein; CVD: cardiovascular disease; cMRI: contrast-enhanced magnetic resonance imaging; DAS28: Disease Activity Score in 28 joints; DTI: diastolic time index; ESR: erythrocyte sedimentation rate; MBP: mean blood pressure; PP: pulse pressure; RA: rheumatoid arthritis; SEVR: subendocardial viability ratio; TTI: tension time index; TNF: tumor necrosis factor.

## Competing interests

The authors declare that they have no competing interests.

## Authors' contributions

AS and ADP participated in the design of the study, recruited patients, performed assessments of pulse wave analysis, conducted data analysis and drafted the manuscript. JH performed statistical analysis of longitudinal data. JPS conducted laboratory analysis. EZ, PPS and GK participated in the design of the study and helped with writing the manuscript. All authors read and approved the final manuscript.
